# Taraxacum officinale extract ameliorates dextran sodium sulphate‐induced colitis by regulating fatty acid degradation and microbial dysbiosis

**DOI:** 10.1111/jcmm.14686

**Published:** 2019-09-29

**Authors:** Wei Chen, Huining Fan, Rui Liang, Rui Zhang, Jing Zhang, Jinshui Zhu

**Affiliations:** ^1^ Department of Gastroenterology Shanghai Jiao Tong University Affiliated Sixth People's Hospital Shanghai China

**Keywords:** cytokines, experimental colitis, fatty acid degradation, microbial dysbiosis, taraxacum officinale extract

## Abstract

Numerous data show that taraxacum officinale extract (TOE) exerts protective effects on inflammatory diseases. However, the underlying mechanisms by which TOE affects dextran sulphate sodium (DSS)‐induced colitis remain unclear. After DSS‐induced colitis were treated with different concentrations of TOE for 8 days, the bodyweight, disease activity index (DAI), colon lengths and pathological scoring were assessed, and histopathological examination was confirmed by HE staining. Furthermore, a transcriptome sequencing was performed by using the colon tissues between TOE and DSS groups, and the differentially expressed genes were conducted for the Kyoto Encyclopaedia of Genes and Genomes (KEGG) and gene set enrichment analysis (GSEA) and were validated by qRT‐*PCR* and immunohistochemistry analysis. In addition, a 16S rDNA sequencing was carried out to distinguish the differential gut microbiota by using the mouse faecal samples between TOE and DSS groups. We found that TOE attenuated the clinical symptoms, lowered the inflammatory scoring and inhibited the secretion of proinflammatory factors TNF‐α, IL‐1β and IL‐6 in DSS‐induced colitis. KEGG and GSEA analysis demonstrated that fatty acid degradation and cytokine‐receptor signalling were predominantly enriched in TOE‐treated colitis as compared with the DSS group. Further investigations revealed that TOE increased the expression levels of Adh5, Aldh3a2 and Acox3, but decreased those of CCL20, CCR6 and CXCL1/5 in DSS‐induced colitis, where TOE also induced the enrichment of *S24‐7* and *adlercreutzia*, but decreased the amount of *anaerostipes, enterococcus, enterobacteriaceae* and *peptostreptococcacea*e. In conclusion, TOE ameliorated DSS‐induced colitis by regulating fatty acid degradation and microbial dysbiosis.

## 1 INTRODUCTION

Acute colitis is characterized by infiltration of inflammatory cells into the mucosa, leading to submucosal congestion and oedema,^1^ and the inflammation cells may involve the whole colon or be limited to colonic segments.^1^ The main symptoms of acute colitis include acute pain, vomiting, weight loss, diarrhoea and bloody stool,^2^ and its incidence and prevalence are being increasing in developing and developed countries.^3^, ^4^ Quinolones and probiotics are commonly used to improve the acute colitis; however, the options for medical management and colitis treatment are limited. Therefore, discovery of cost‐effective and efficacious agents for colitis is necessary.

Taraxacum officinale (TO), a herbaceous perennial plant of the family Asteraceae, is widely used as an herbal remedy in Asia, Europe and North America. The therapeutic value of TO was mentioned in acute mastitis in 659 A. D in China, and it was used to cure liver and spleen ailments by the Arabian physicians during the 10th and 11th centuries. Accumulating evidence indicates that taraxacum officinale extract (TOE) exhibits various biological activities, such as anti‐inflammatory,^5^, ^6^ antioxidant 7, 8 and antifibrotic activities,^9^, ^10^ and possess the properties against type 2 diabetes,^11^ non‐alcoholic fatty liver disease (NAFLD),^12^, ^13^ obesity 14 and cancers.^15^, ^16^ However, there is little knowledge about the effects of TOE on acute colitis.

Chemistry‐induced experimental colitis models are widely applied owing to the fast onset of inflammation and relatively simple operation procedures.^17^, ^18^ Dextran sulphate sodium (DSS) is used to induce severe colitis in mice, characterized by weight loss, bloody diarrhoea, ulcer formation, loss of epithelial cells and infiltrations with neutrophils,^19^ which is associated with DSS‐caused toxicity towards gut epithelial cells and the integrity of mucosal barrier.^20^ Moreover, DSS tends to decrease intestinal microbial community evenness 21 and enhances mucosal CD4^+^ T responses, involved in the pathogenesis of acute colitis.^22^

In the present study, we assessed the effects of TOE on DSS‐induced colitis in mice and found that TOE attenuated DSS‐induced colitis by regulating fatty acid degradation, cytokine‐receptor signalling and microbial dysbiosis. Our findings might provide a novel strategy for the treatment of acute colitis.

## 2 METHODS AND MATERIALS

### 2.1 Experimental Animals

Male C57BL/6 mice, 6‐8 weeks old and weighing 18 ± 2 g were provided by West Pui Kai experimental animal Co., Ltd and fed in SPF standard laboratory conditions at animal laboratory centre of our hospital. The animal study was approved by the ethics committee of Shanghai Sixth People's Hospital (No: 2018‐0080).

### 2.2 DSS‐induced colitis and TOE treatment

According to the previous report,^23^ acute experimental colitis was induced by drinking 3% (wt/vol) DSS (36‐50 kD, MP Biomedicals) for a week and then was treated by the low‐dose TOE (TOEL, 0.9 g/kg/d) or high‐dose TOE (TOEH, 1.8 g/kg/d) for 8 days. The mice were classified into control group (distilled water), DSS group, TOEL and TOEH groups.

### 2.3 Tissue collection

After the mice were sacrificed, the colon tissues were collected. The length of each colon was recorded, and each colon was washed in PBS. Then, 0.5 cm colon segments near the anus were used for RNA extraction and 1.0 cm were fixed in 4% formaldehyde for histological analysis. The remaining tissues were rapidly frozen in liquid nitrogen at −80°C.

### 2.4 Clinical scoring of murine colitis

According to the criteria described by Stefan Wirtz,^23^ the mice in each group was observed daily in the morning, and the weight loss, stool consistency and the degree of intestinal bleeding were recorded. Criteria for the different scores are shown in Table S1. A disease activity index (DAI) was calculated based on the sum of the scores for bodyweight loss, diarrhoea and bleeding.

### 2.5 Haematoxylin and eosin (H&E) staining

The colon tissues were isolated from the mice in each group and fixed on a 4% paraformaldehyde solution for 48 hours and embedded in paraffin. Histological examinations were performed by H&E staining. According to the previous studies,^23^, ^24^ the pathological scoring was conducted.

### 2.6 Real‐time PCR (RT‐PCR)

According to the instructions, total RNA was isolated from colon tissues using the Trizol reagent (Invitrogen). Complementary DNA (cDNA) is produced by RNA reverse transcription by using the PrimeScript™ Reverse Transcription Kit (TakaRa). The procedures were performed as follow: Stage 1:Pre‐denaturation at 95°C for 30 seconds, 1 cycle; Stage 2: PCR, at 95°C for 5 seconds and 60°C for 34 seconds, 40 cycles; and Stage 3: at 95°C for 30 seconds and 60°C for 1 minute, 1 cycle. The gene expression levels were calculated using the 2^−ΔΔCt^ method. Glyceraldehyde 3‐phosphate dehydrogenase (GAPDH) was used as the internal control. All qPCR reactions were performed in duplicate. The primers used in this study were listed in Table 1.

**Table 1:** List of the gene primers

Gene	Forward primer	Reverse primer
GAPDH	CCTCGTCCCGTAGACAAAATG	TGAGGTCAATGAAGGGGTCGT
TNF‐α	CCCTCACACTCACAAACCACC	CTTTGAGATCCATGCCGTTG
IL‐6	GAAATGATGGATGCTACCAAACTG	GACTCTGGCTTTGTCTTTCTTGTT
IL‐1β	CACTACAGGCTCCGAGATGAAC	TCCATCTTCTTCTTTGGGTATTGC
IFN‐γ	AGCAACAACATAAGCGTCAT	CCTCAAACTTGGCAATACTC
CCL20	CAGGCAGAAGCAAGCAACTACG	TGACTCTTAGGCTGAGGAGGTTC
CXCL5	GCGTTGTGTTTGCTTAACCGT	AGCTTTCTTTTTGTCACTGCCC
CD40	ACCCATGTGACTCAGGCGAAT	TGGTGCAGTGTTGTCCTTCC
CXCL1	CTGCACCCAAACCGAAGTCAT	TGGGGACACCTTTTAGCATCT
CCR6	CCTCCTGGGCAACATTATGGT	GCATCGCTGAAAACCCAAGTG
Adh5	ACAGATGGGGGCGTGGATTA	GCCTTTCCATGTGCGTCCT
Acox3	GACGCATCTCCATCATCAGCAT	GCCAGAGCATGGATCTCACGT
Tnfsf11	CCATCGGGTTCCCATAAAGTCA	CAGTTTTTCGTGCTCCCTCCTT
CXCL13	GGCCACGGTATTCTGGAAGC	TTGGCACGAGGATTCACACA
Tnfrsf8	CGGGAACTCTCCTCGAATCTGT	ATGGCCTGAGGAGTGGCAT
Aldh3a2	GCGAAGCCTCCCTCCAGAAT	CCTCATCCATCTCTCCACCGAA
CCL6	AGAAGATCGTCGCTATAACCCTC	TAGGCACCTCTGAACTCTCCG
CCl28	CTCACCTGAGTCATTGCCAGA	CATGGGAAGTATGGCTTCTGAG
CCL5	TGCCCACGTCAAGGAGTATTT	GATGTATTCTTGAACCCACTTCTTC

### 2.7 Immunohistochemistry (IHC)

To assess the severity of DSS‐induced colitis, the intensity of inflammation and other markers related to fatty acid degradation and cytokines were determined by using IHC analysis. The colon tissues were immune‐stained for anti‐TNF alpha (17590‐1‐ap; Proteintech), anti‐IL‐6 (21865‐1‐AP, Proteintech), anti‐IL‐1β (ab33591, Lianke Biotechnology), anti‐ACOX3 (NBP1‐85901, Novus Biologicals), anti‐ALDH3A2 (15090‐1‐AP, Proteintech), anti‐CXCL5 (ab9802, Abcam), anti‐GRO alpha ( also termed as anti‐CXCL1, ab86436, Abcam), anti‐MIP3a ( also termed as anti‐CCL20, ab136904, Abcam) and anti‐ADH5 (ab177932, Abcam) as previously described.^25^

### 2.8 Transcriptome sequencing analysis

Total RNA was isolated from the colon tissues of the control, DSS, TOEL and TOEH groups and then was detected by agarose gel electrophoresis. NanoDrop ND‐1000 was used for further quality control of total RNA. A total of 1‐2 μg RNA from each sample was used as input material for generation of the RNA library. Following cluster generation, the libraries were sequenced on an Illumina Hiseq 4000 platform. After the transcriptome sequencing, the raw data were subjected to the KEGG and GSEA analysis.

### 2.9 16S rDNA sequencing

Bacterial genomic DNA was extracted from stool samples in each group. The 16S rDNA V4 region was amplified by PCR using barcoded Illumina adapter‐containing 515F and 806R primers. Qubit 3.0 was used to quantify each sample, and pooling of equal quality was used as a library to ensure the homogeneity of samples. After the library was qualified, illumina high‐throughput sequencing platform (HiSeq/MiniSeq) was used for 16S rDNA sequencing.

### 2.10 Statistical analysis

Statistical analyses were conducted by SPSS 17.0 (IBM, SPSS) and GraphPad Prism. Data are expressed as mean ± SD. Analysis of Variance (ANOVA) was used to analyse the difference between four groups, and independent *t* test was used to analyse the significance of two groups. *P* < .05 was considered statistically significant.

## 3 RESULTS

### 3.1 TOE attenuated the severity of clinical symptoms and inflammatory infiltration in DSS‐induced colitis

**Figure 1:**
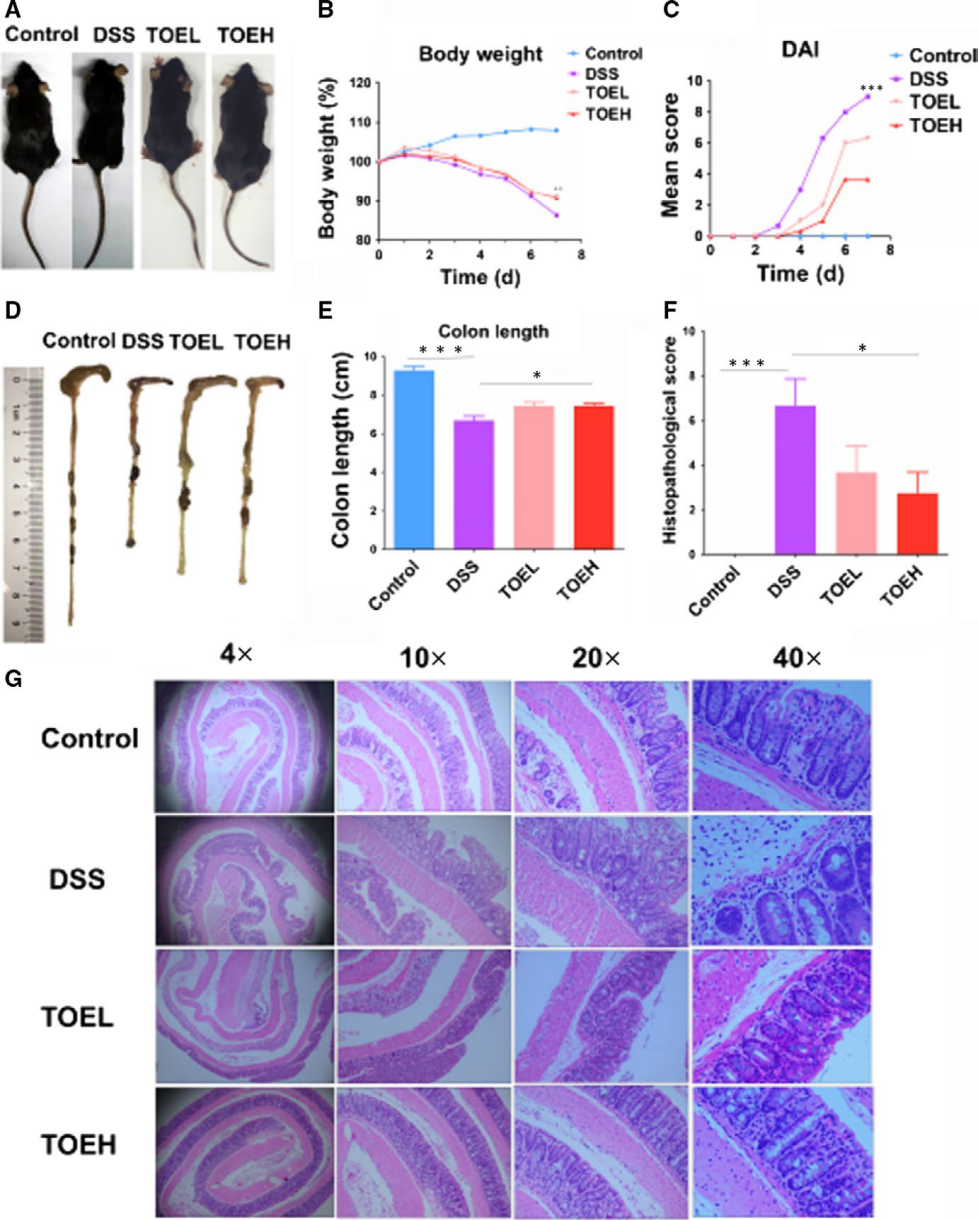
TOE attenuated the severity of clinical symptoms and inflammatory infiltration in DSS‐induced colitis. A, Representative schematic of the mice in control, DSS, TOEL and TOEH groups. B, Comparison of the mean bodyweight in these four groups (*P < .05, DSS vs TOEH; **P < .01, DSS vs TOEL). C, Comparison of the alterations in disease activity index (DAI; ***P < .001, DSS vs TOEH or TOEL). D. Representative schematic of the colon samples in control, DSS, TOEL and TOEH groups. E. Comparison of the mean colon lengths (***P < .001, control vs DSS; *P < .05, DSS vs TOEH). F, H&E analysis of the histopathological scoring in colon tissue samples from these four groups (4×, 10×, 20×, 40×). G, Comparison of the histopathological scores in colon tissue samples from these four groups. (***P < .001, control vs DSS; *P < .05, DSS vs TOEH)

To determine the effects of TOE on acute colitis, we established a DSS‐induced acute colitis model. Representative schematic of the mice in four groups was demonstrated in Figure 1A. The bodyweight was dramatically decreased by DSS as compared with the control group, and this result could be reversed by TOEH and TOEL (Figure 1B). Likewise, an increased cumulative DAI was increased by DSS as compared with the control group, but this effect was reversed by the TOEH and TOEL (Figure 1C). Representative schematic of the colon tissues in four groups was indicated in Figure 1D and the shortened colon length was caused by DSS as compared with the control group, but this effect was reversed by TOEH rather than TOEL (Figure 1E). Additionally, HE staining revealed that DSS induced an obvious inflammatory response, characterized by neutrophil infiltration, crypt loss, submucosal oedema and goblet cell loss, but TOEH and TOEL reduced these inflammatory responses (Figure 1F). Histopathological scores were substantially increased in DSS group as compared with the control group, but these results were reversed by TOEH rather than TOEL (Figure 1G).

### 3.2 TOE diminished the production of pro‐inflammatory cytokines in DSS‐induced colitis

**Figure 2:**
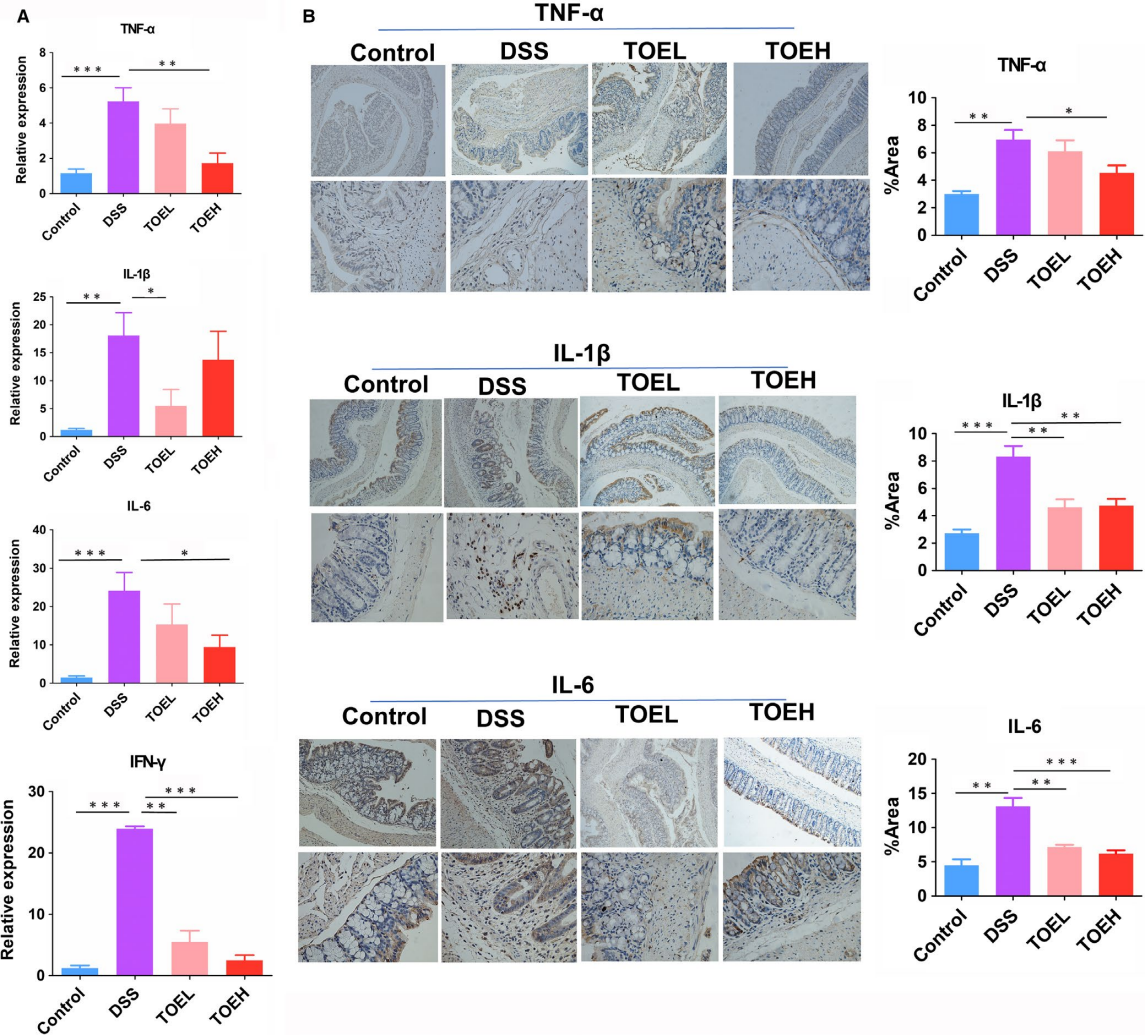
TOE reduced the production of inflammatory cytokines in DSS‐induced colitis. A, B, RT‐PCR and IHC analysis of the expression levels of inflammatory cytokines TNF‐*α*, IL‐6, IL‐1*β *and IFN**‐*γ*** in colon tissue samples from these four samples*.* *P < .05, **P < .01, ***P < .001

The pro‐inflammatory cytokines act a critical role in the pathogenesis of acute colitis, and the levels of pro‐inflammatory cytokines are reported increased in DSS‐induced colitis.^26^ To determine whether TOE exerts anti‐inflammatory effects in DSS‐induced colitis, we examined the expression levels of pro‐inflammatory cytokines TNF‐α, IL‐6, IFN‐γ and IL‐1β in colon tissues by RT‐PCR (Figure 2A) and IHC analysis (Figure 2B), which showed that their expression levels were notably elevated by DSS as compared with the control group, but these effects were reversed by TOEH and (or) TOEL.

### 3.3 TOE promoted the fatty acid degradation in DSS‐induced colitis

**Figure 3:**
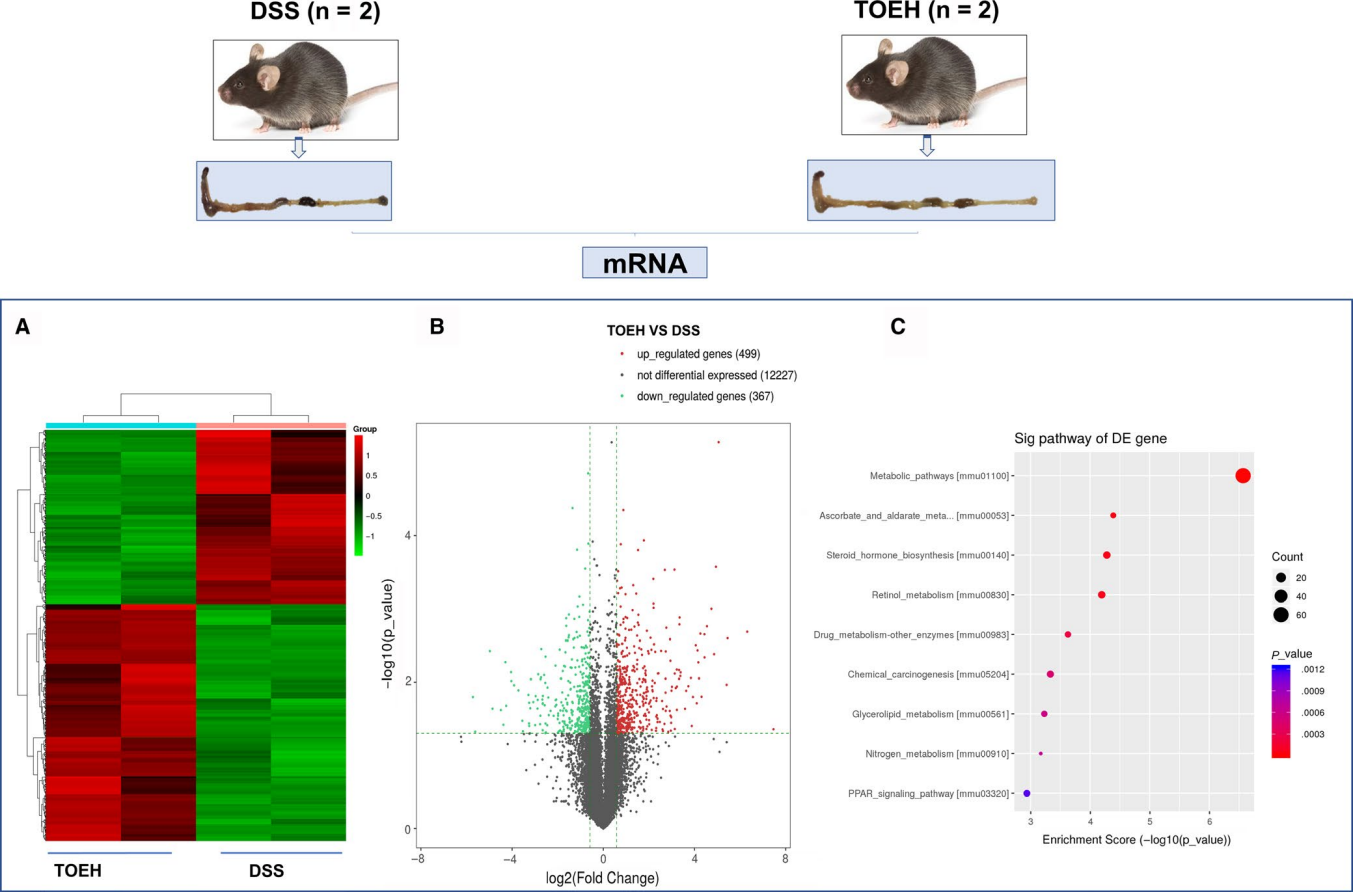
The differentially expressed genes were identified between DSS and TOEH groups by transcriptome sequencing. A, Hierarchical clustering analysis was performed to establish the gene expression profiling between these two groups. B, Volcano plotting of the differentially expressed genes between DSS and TOEH groups. Red colours indicated the up‐regulated genes and blue colours indicated the down‐regulated genes. C, The genes that were most significantly differentially expressed were analysed by KEGG enrichment analysis

**Figure 4:**
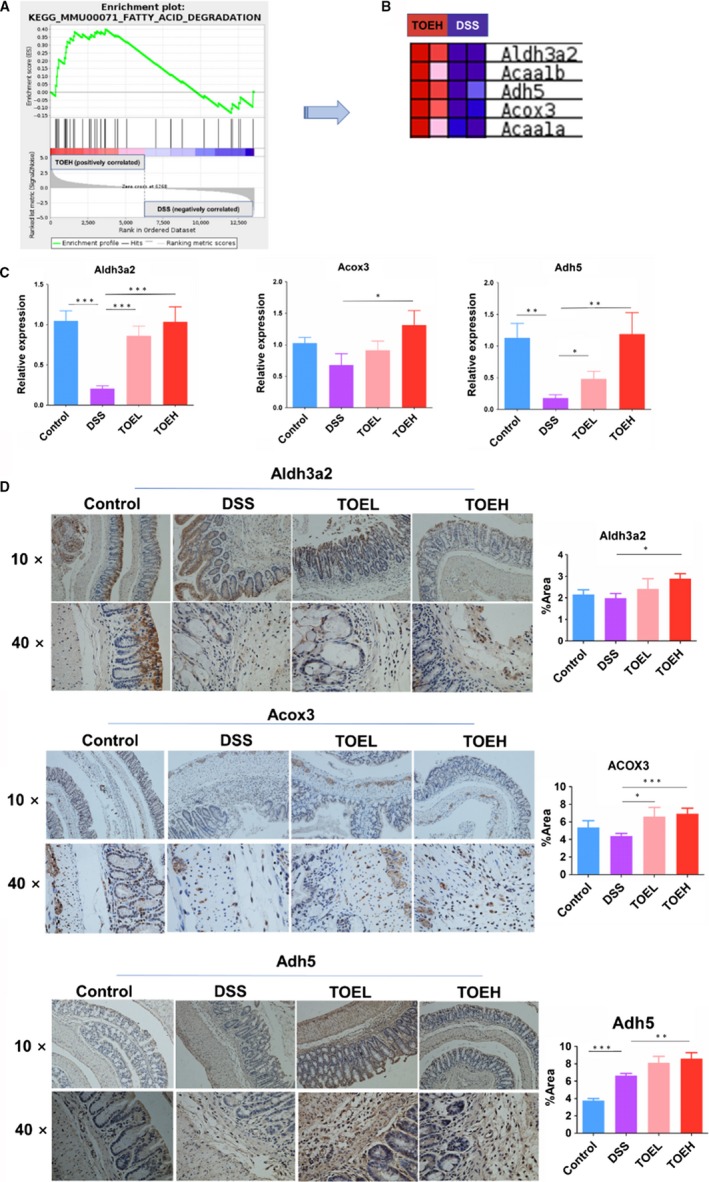
TOE promoted the fatty acid degradation in DSS‐induced colitis. A, B, GSEA identification of the five major genes in fatty acid degradation in TOEH group as compared with the DSS group. C, D, RT‐PCR and IHC analysis of the expression levels of Aldh3a2, Acox3 and Adh5 in TOEH and DSS groups. *P < .05, **P < .01, ***P < .001

To understand the underlying mechanisms by which TOE attenuated DSS‐induced colitis, we performed a transcriptome sequencing using the colon tissues between the control, DSS, TOEL and TOEH groups. Based on the fold change, the differentially expressed genes were identified by thermogram and volcano map between control, DSS and TOEH groups (Figure S1A,B and Figure 3A,B), of which 499 up‐regulated genes and 367 down‐regulated genes were identified between DSS and TOEH groups (Figure 3A,B). KEGG and GSEA analysis unveiled that fatty acid degradation, a key fat metabolic pathway, was indicated to be enriched in TOEH treatment group as compared with the DSS group (Figure S1C,D and Figure 4A). Five major genes in fatty acid degradation were shown to be induced by TOEH, of which Aldh3a2, Acox3 and Adh5 displayed the significantly increased expression in TOEH group as compared with the DSS group (Figure 4B). Further RT‐PCR (Figure 4C) and IHC analysis (Figure 4D) confirmed that the expression levels of Aldh3a2, Acox3 and Adh5 were lowered by DSS as compared with the control group, but were increased by TOEH and (or) TOEL as compared with the DSS group.

### 3.4 TOE inhibited the cytokine‐receptor signalling in DSS‐induced colitis

**Figure 5:**
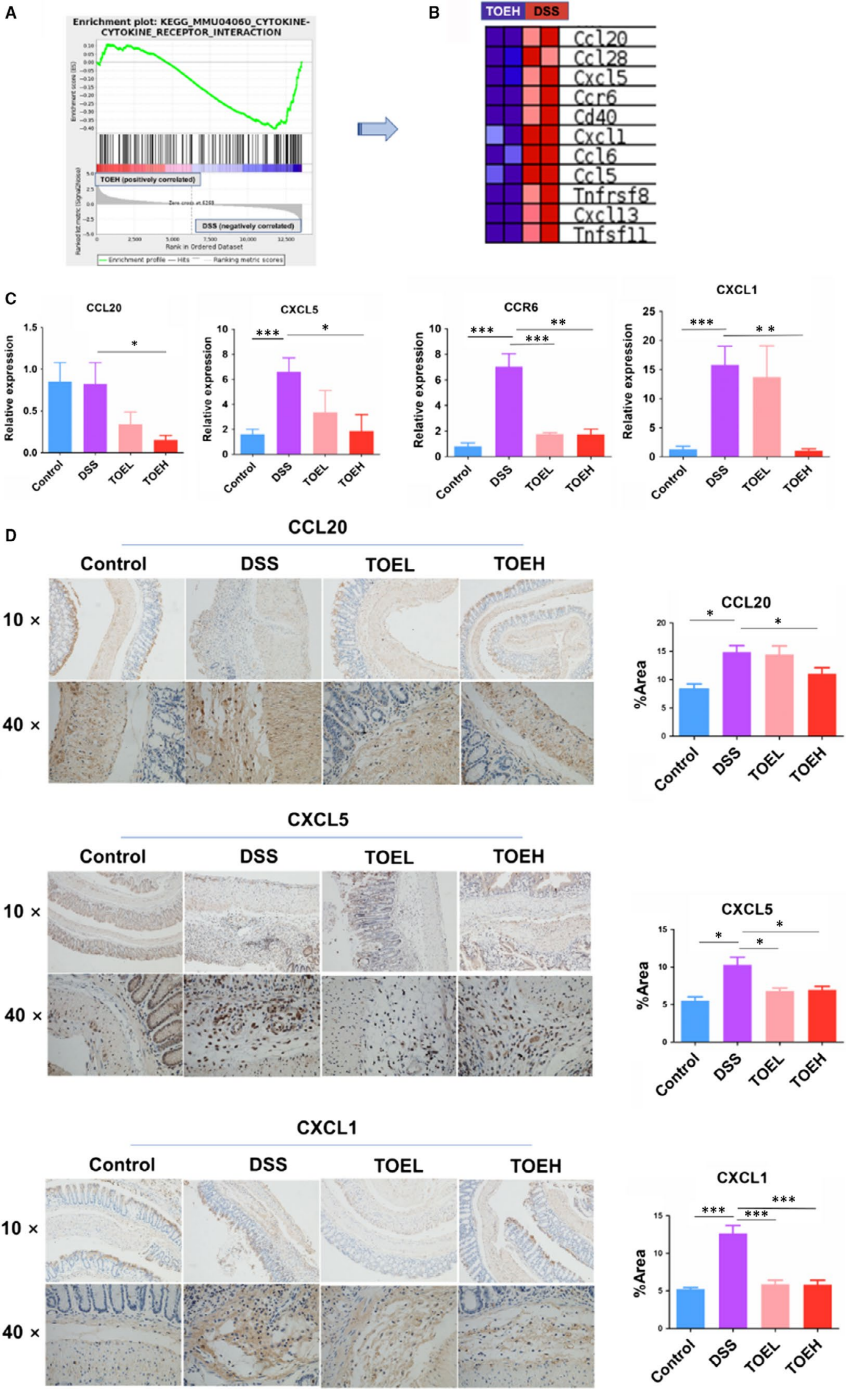
TOE inhibited the cytokine‐receptor signalling in DSS‐induced colitis. A, B, GSEA identification of the 11 major genes in cytokine‐receptor signalling in TOEH group as compared with the DSS group. C, D, RT‐PCR and IHC analysis of the expression levels of CCL20, CXCL5, CCR6 and CXCL1 in TOEH and DSS groups. *P < .05, **P < .01, ***P < .001

Further GSEA analysis showed that the cytokine‐receptor signalling was involved in TOEH‐treated colitis as compared with the DSS group (Figure 5A), and 11 genes in this signalling were identified to be down‐regulated in TOEH treatment group, of which 11 genes (CCL20, CCL28, CXCL5, CCR6, CXCL1, CXCL13, CCL6, CCL5, CD40, Tnfsf11 and Tnfrsf8) had the markedly decreased expression in TOEH group as compared with the DSS group (Figure 5B). RT‐PCR (Figure 5C) and IHC analysis (Figure 5D) verified that the expression levels of CCL28, CCL6, CCL5, CXCL5, CCR6, CXCL1, CD40, Tnfsf11 and Tnfrsf8 were increased by DSS as compared with the control group (Figure 5C,D and Figure S2), but TOEH and (or) TOEL counteracted DSS‐induced CCL20, CXCL5, CCR6, CXCL1 and CXCL13 expression in colon tissues (Figure 5C,D and Figure S2).

### 3.5 TOE regulated the gut microbial dysbiosis in DSS‐induced colitis

**Figure 6:**
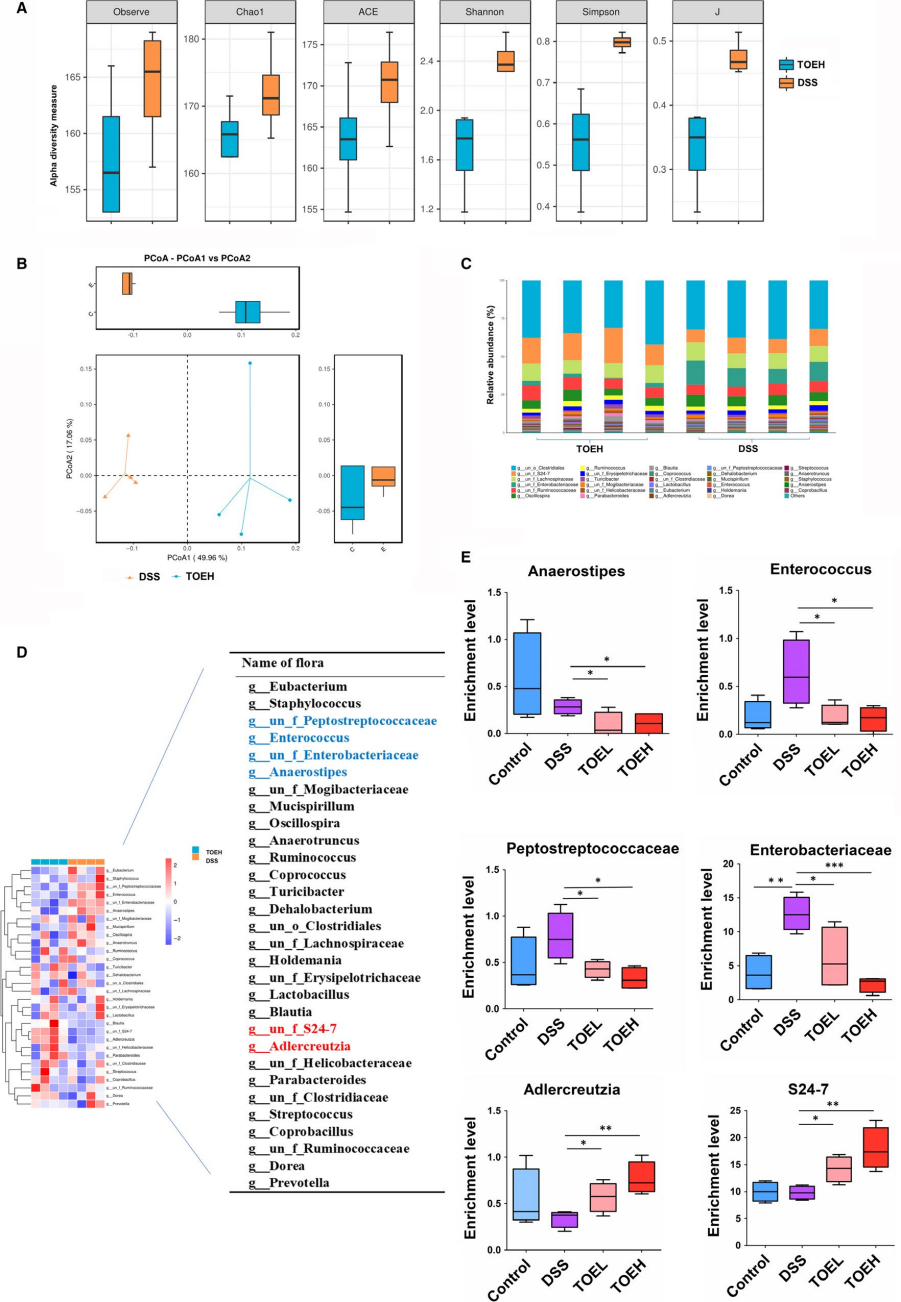
TOE regulated the gut microbial dysbiosis in DSS‐induced colitis. A, Alpha diversity was estimated by the Observe, Chao1, ACE, Shannon, Simpson and J index in DSS and TOEH groups. B, Beta diversity was assessed by the weighted PCoA in DSS and TOEH groups. C, Comparison of the difference in genus levels of gut microbiota between DSS and TOEH groups. D, The heat maps of the top 30 gut microbiota species in DSS and TOEH groups. E, Bar plots showed the relative abundance in genus levels in DSS and TOEH groups. *P < .05, **P < .01, ***P < .001

Gut microbiota is associated with the acute colitis 21, and whether TOE modifies the gut microbiota in DSS‐induced colitis was further assessed using the colon stool samples and 16S rDNA sequencing between the four groups. The alpha diversity of microbial communities, as indicated by the Observe, Chao1, ACE, Shannon, Simpson and J index, tended to increase in DSS‐induced colitis, but was decreased by TOEH (Figure 6A). In addition, beta diversity had a significant difference between DDS and TOEH groups based on the weighted PCoA (Figure 6B). Moreover, the genus abundant levels showed that *Clostridiale, S24‐7, Lanchnospiraceae, Enterobacteriaceae, Ruminococcaceae* and *Oscillospira* had a significant difference between DSS and TOEH groups (Figure 6C). The thermograms showed that the top 30 microflora were found between DSS and TOEH groups (Figure 6D). As shown in Figure 6E, the enrichment levels of these six bacteria displayed a statistical difference, of which the amount of *anaerostipes, enterococcus, peptostreptococcaceae* and *enterobacteriaceae* was lowered in TOEH group, but that of *S24‐7 and adlercreutzia* was elevated as compared with that in DSS group. The alpha and beta diversity, and the genus abundant levels between DSS and Control groups were indicated in Figure S3.

## 4 DISCUSSION

**Figure 7:**
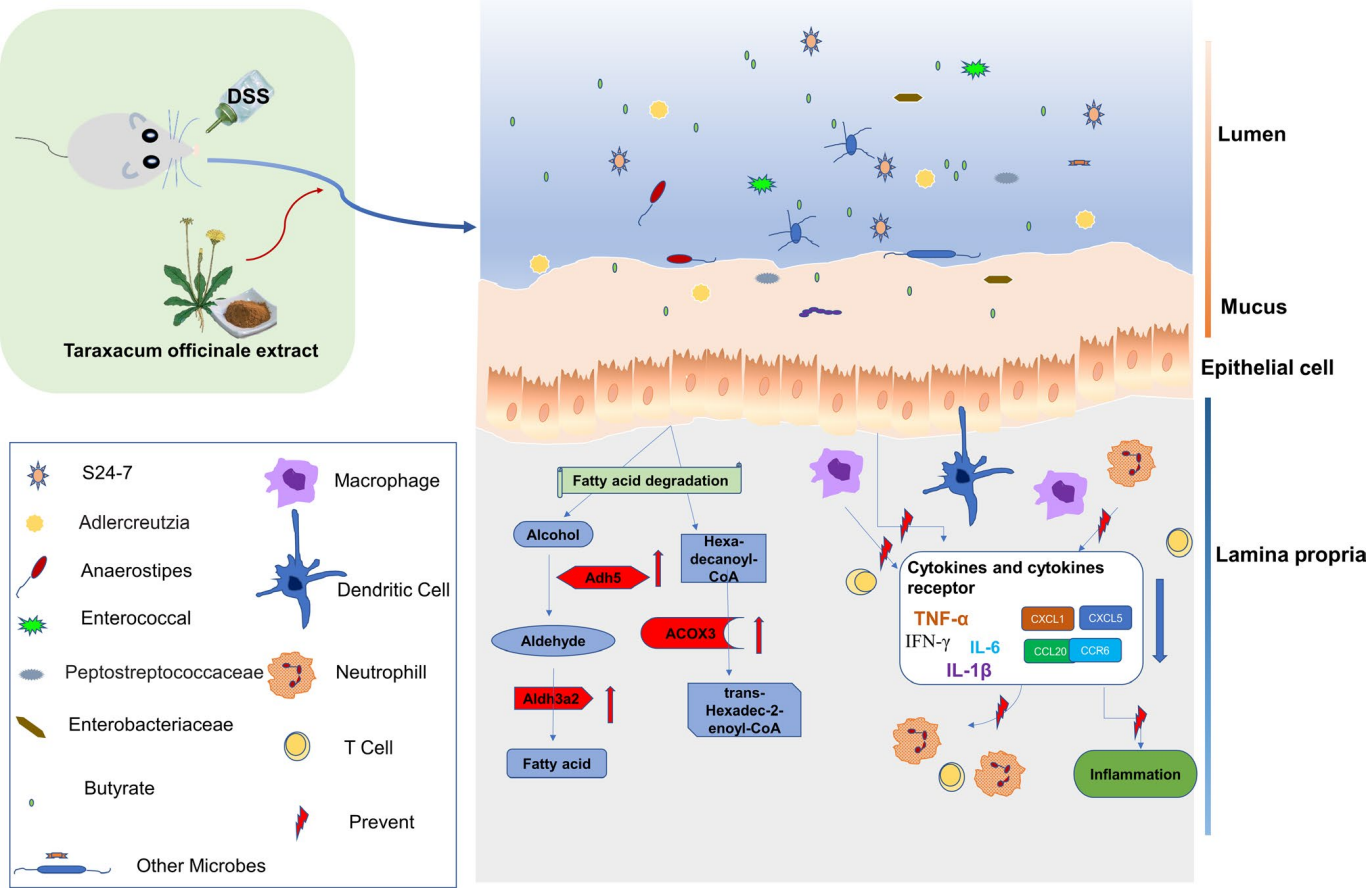
Schematic representation of the proposed mechanism of TOE on DSS‐induced colitis. DSS destroyed the integrity of mucosal barrier in mice and induced colonic inflammation. But TOE attenuated the clinical symptoms, lowered the inflammatory scoring and inhibited the secretion of pro‐inflammatory factors (TNF‐α, IL‐1β and IL‐6) in DSS‐induced colitis. TOE also increased the expression levels of Adh5, Aldh3a2 and Acox3, but down‐regulated the expression of CCL20, CCR6 and CXCL1/5 in DSS‐induced colitis. In addition, TOE regulated microbial dysbiosis in DSS‐induced colitis

In the present study, TOE displayed a protective effect on DSS‐induced colitis by improving the severity of clinical symptoms and inflammatory responses. Transcriptome sequencing and GSEA analysis revealed that fatty acid degradation and cytokine‐receptor signalling were implicated in TOE‐treated colitis as compared with the DSS group. RT‐PCR and IHC analysis validated that TOE up‐regulated the expression of Adh5, Aldh3a2 and Acox3, but down‐regulated the expression of CCL20, CXCL5, CCR6 and CXCL1 in DSS‐induced colitis. Gut microbiota analysis showed that TOE promoted the enrichment of S24‐7 and adlercreutzia, but decreased the amount of anaerostipes, enterococcus, enterobacteriaceae and peptostreptococcaceae (Figure 7).

Acute colitis is a common idiopathic disease characterized by intestinal epithelial barrier damage and inflammatory homoeostasis damage, leading to the secretion of pro‐inflammatory cytokines, such as TNF‐α, IFN‐γ, IL‐1β and IL‐6,^27^ associated with DSS‐induced colitis.^28^ Herein, we found that TOE relieved the severity of DSS‐induced colitis and inflammatory infiltration and decreased the production of cytokines TNF‐α, IL‐1β and IL‐6, suggesting that TOE might exhibit an anti‐inflammatory activity in DSS‐induced colitis.

Furthermore, GSEA analysis identified that fatty acid degradation was involved in TOE‐treated colitis. Alcohol dehydrogenase 5 (Adh5), a key enzyme of alcohol dehydrogenase family acts by regulating GSNO, nitric oxide (NO) and RNS.^29^, ^30^ Adh5 is involved in smooth muscle relaxation, immune response, inflammation and oncogenesis,^31^ and alters the cellular homoeostasis.^32^ Acyl‐CoA oxidase 3 (Acox3), a crosstalk enzyme between alpha‐linolenic acid metabolism and fatty acid metabolism, participates in the desaturation of 2‐methyl branched‐chain fatty acids in peroxisomes and inhibits the progression of cervical cancer.^33^ Aldehyde dehydrogenase (Aldh) isozymes act in the detoxification of aldehydes. Aldh3a2 can code fatty aldehyde dehydrogenase in mammal,^34^, ^35^ and pathogenic variants of Aldh3a2 lead to Sjögren‐Larsson syndrome.^36^ Herein, we confirmed that TOE exhibited a promoting effect on the fatty acid degradation by increasing Acox3, Adh5 and Aldh3a2 expression in DSS‐induced colitis.

GSEA analysis also identified that cytokine‐receptor signalling was associated with TOE‐treated colitis. CCL20 can be induced by inflammation in endothelial cells, monocytes and dendritic cells,^37^ and CCL20 and its receptor CCR6 are associated with inflammatory bowel disease (IBD).^38^, ^39^ CXC chemokines CXCL1/5 can be produced by colon epithelial cells 40, 41 and participate in inflammatory response by recruiting neutrophil in colitis.^42^ Herein, TOE had the protective effects on DSS‐induced colitis by repressing the production of cytokines CCL20, CXCL1/5 and CCR6.

Gut microbiota is separated from the host compartment by a single layer of epithelial cells in response to the threats from commensals.^43^, ^44^ Loss of the intestinal barrier causes the autoimmune and inflammatory diseases,^45^, ^46^ and intestinal microbiota enhances the barrier function by promoting the homoeostasis of mucosal immunity.^47^ The dysbiosis of gut microbiome is associated with the colitis.^48^, ^49^, ^50^, ^51^ Herein, a 16S rDNA sequencing showed that *S24‐7, adlercreutzia, anaerostipes, enterococcus, enterobacteriaceae* and *peptostreptococcaceae* had a significant difference between TOEH and DSS groups. The enrichment levels of *Bacteroidales S24‐7* and *adlercreutzia* are decreased, but those of *enterobacteriaceae* are increased in IBD.^52^, ^53^ *Anaerostipes* can produce butyrate from acetic and lactic acids, which maintains the intestinal barrier and exerts anti‐inflammatory properties.^54^ Increased abundance of *peptostreptococcaceae* is associated with ulcerative colitis,^55^ colorectal cancer 56 and NAFLD.^57^ In accordance with these studies, we found that TOE promoted the enrichment of *S24‐7* and *adlercreutzia*, but reduced the amount of *anaerostipes, enterococcus, enterobacteriaceae* and *peptostreptococcaceae*.

In conclusion, our findings demonstrated that TOE attenuated DSS‐induced colitis by regulating fatty acid degradation and microbial dysbiosis, and these findings might provide novel insights into the molecular mechanisms and therapeutic strategies for acute colitis.

## CONFLICTS OF INTEREST

Authors declare that they do not have any conflict of interest.

## AUTHORS' CONTRIBUTIONS

Jinshui Zhu and Jing Zhang designed this study and Wei Chen drafted the manuscript. Wei Chen, Huining Fan, Rui Liang and Rui Zhang performed the experiments. Huining Fan and Rui Liang conducted the statistical analysis. Wei Chen wrote the paper and Jing Zhang revised the paper. All authors read and approved the final manuscript.

## Data Availability

All data used to support the findings of this study are available from the corresponding authors upon request.

## References

[1] Barral M , Boudiaf M , Dohan A . MDCT of acute colitis in adults: an update in current imaging features. Diagn Interv Imaging. 2015;96(2):133‐149. 24835625 10.1016/j.diii.2014.04.008

[2] Lombardi V , Etcheverría I , Carrera I . Prevention of Chronic experimental colitis induced by dextran sulphate sodium (DSS) in mice treated with FR91. J Biomed Biotechnol. 2012;2012:1‐9. 22619498 10.1155/2012/826178 PMC3348609

[3] Khoon NW , Wong SH , Ng SC . Changing epidemiological trends of inflammatory bowel disease in Asia. Intest Res. 2016;14(2):111. 27175111 10.5217/ir.2016.14.2.111 PMC4863044

[4] Molodecky NA , Soon IS , Rabi DM . Increasing incidence and prevalence of the inflammatory bowel diseases with time. based on systematic review. Gastroenterology. 2011;142(1):46‐54.e42. 22001864 10.1053/j.gastro.2011.10.001

[5] Jeon D , Kim SJ , Kim HS . Anti‐inflammatory evaluation of the methanolic extract of Taraxacum officinale in LPS‐stimulated human umbilical vein endothelial cells. BMC Complement Altern Med. 2017;17(1):508. 29187173 10.1186/s12906-017-2022-7 PMC5707789

[6] Yang HJ , Kim MJ , Kwon DY . Gastroprotective actions of, Taraxacum coreanum, Nakai water extracts in ethanol‐induced rat models of acute and chronic gastritis. J Ethnopharmacol. 2017;208:84‐93. 28687507 10.1016/j.jep.2017.06.045

[7] You Y , Yoo S , Yoon HG . In vitro and in vivo hepatoprotective effects of the aqueous extract from Taraxacum officinale (dandelion) root against alcohol‐induced oxidative stress. Food Chem Toxicol. 2010;48(6):1632‐1637. 20347918 10.1016/j.fct.2010.03.037

[8] Belén G‐C , Raquel FD , Dávalos A . In vitro hypolipidemic and antioxidant effects of leaf and root extracts of taraxacum officinale. Med Sci. 2015;3(2):38‐54. 10.3390/medsci3020038 PMC5635758 29083390

[9] Domitrović R , Jakovac H , Romić Z . Antifibrotic activity of Taraxacum officinale root in carbon tetrachloride‐induced liver damage in mice. J Ethnopharmacol. 2010;130(3):569–577. 20561925 10.1016/j.jep.2010.05.046

[10] Du J , Liang Z , Xu J , et al. Plant‐derived phosphocholine facilitates cellular uptake of anti‐pulmonary fibrotic HJT‐sRNA‐m7. Sci China Life Sci. 2019;62(3):309‐320. 28378154 10.1007/s11427-017-9026-7

[11] Wirngo FE . The physiological effects of dandelion (Taraxacum officinale) on type 2 diabetes. Rev Diabet Stud. 2016;13(2–3):113. 28012278 10.1900/RDS.2016.13.113 PMC5553762

[12] Davaatseren M , Hur HJ , Yang HJ , et al. Taraxacum official (dandelion) leaf extract alleviates high‐fat diet‐induced nonalcoholic fatty liver. Food Chem Toxicol. 2013;58(3):30‐36. 23603008 10.1016/j.fct.2013.04.023

[13] Davaatseren M , Hur HJ , Yang HJ . Dandelion leaf extract protects against liver injury induced by methionine‐ and choline‐deficient diet in mice. J Med Food. 2013;16(1):26. 23256442 10.1089/jmf.2012.2226

[14] González‐Castejón M , García‐Carrasco B , Fernández‐Dacosta R . Reduction of adipogenesis and lipid accumulation by Taraxacum officinale (Dandelion) extracts in 3T3L1 adipocytes: an in vitro study. Phytother Res. 2014;28(5):745‐752. 23956107 10.1002/ptr.5059

[15] Rehman G , Hamayun M , Iqbal A . Effect of methanolic extract of dandelion roots on cancer cell lines and AMP‐activated protein kinase pathway. Front Pharmacol. 2017;8:875. 29234282 10.3389/fphar.2017.00875 PMC5712354

[16] Zhu H , Zhao H , Zhang L . Dandelion root extract suppressed gastric cancer cells proliferation and migration through targeting lncRNA‐CCAT1. Biomed Pharmacother. 2017;93:1010‐1017. 28724210 10.1016/j.biopha.2017.07.007

[17] Strober W , Fuss IJ , Blumberg RS . The immunology of mucosal models of inflammation. Annu Rev Immunol. 2002;20(undefined):495‐549. 11861611 10.1146/annurev.immunol.20.100301.064816

[18] Perše M , Cerar A . Dextran sodium sulphate colitis mouse model: traps and tricks. J Biomed Biotechnol. 2012;2012(undefined):718617. 22665990 10.1155/2012/718617 PMC3361365

[19] Okayasu I , Hatakeyama S , Yamada M . A novel method in the induction of reliable experimental acute and chronic ulcerative colitis in mice. Gastroenterology. 1990;98(3):694‐702. 1688816 10.1016/0016-5085(90)90290-h

[20] Wirtz S , Neufert C , Weigmann B . Chemically induced mouse models of acute and chronic intestinal inflammation. Nat Protoc. 2007;2(3):541‐546. 17406617 10.1038/nprot.2007.41

[21] Zhang H , Hua R , Zhang B . Serine alleviates dextran sulfate sodium‐induced colitis and regulates the gut microbiota in Mice. Front Microbiol. 2018;9(undefined):3062. 30619148 10.3389/fmicb.2018.03062 PMC6295577

[22] Sang L , Chang B , Zhu J . Dextran sulfate sodium‐induced acute experimental colitis in C57BL/6 mice is mitigated by selenium. Int Immunopharmacol. 2016;39:359‐368. 27533281 10.1016/j.intimp.2016.07.034

[23] Wirtz S , Popp V , Kindermann M . Chemically induced mouse models of acute and chronic intestinal inflammation. Nat Protoc. 2017;12(7):1295‐1309. 28569761 10.1038/nprot.2017.044

[24] Takagawa T , Kitani A , Fuss I . An increase in LRRK2 suppresses autophagy and enhances Dectin‐1–induced immunity in a mouse model of colitis. Sci Transl Med. 2018;10(444):p. eaan8162. 10.1126/scitranslmed.aan8162 PMC6636639 29875204

[25] Huang Y , Zhang J , Hou L . LncRNA AK023391 promotes tumorigenesis and invasion of gastric cancer through activation of the PI3K/Akt signaling pathway. J Exp Clin Cancer Res. 2017;36(1):194. 29282102 10.1186/s13046-017-0666-2 PMC5745957

[26] Bento AF , Leite DF , Marcon R . Evaluation of chemical mediators and cellular response during acute and chronic gut inflammatory response induced by dextran sodium sulfate in mice. Biochem Pharmacol. 2012;84(11):1459‐1469. 23000912 10.1016/j.bcp.2012.09.007

[27] Dionne S , Hiscott J , D'Agata I . Quantitative PCR analysis of TNF‐alpha and IL‐1 beta mRNA levels in pediatric IBD mucosal biopsies. Dig Dis Sci. 1997;42(7):1557‐1566. 9246063 10.1023/a:1018895500721

[28] Rojas‐Cartagena C , Flores I , Appleyard CB . Role of tumor necrosis factor receptors in an animal model of acute colitis. Cytokine. 2005;32(2):85‐93. 16213154 10.1016/j.cyto.2005.08.001

[29] Beckman JS , Koppenol WH . Nitric oxide, superoxide, and peroxynitrite: the good, the bad, and ugly. Am J Physiol. 1996;271(1):1424‐1437. 10.1152/ajpcell.1996.271.5.C1424 8944624

[30] Radi R , Beckman JS , Bush KM . Peroxynitrite oxidation of sulfhydryls. The cytotoxic potential of superoxide and nitric oxide. J Biol Chem. 1991;266(7):4244‐4250. 1847917

[31] Barnett SD , Buxton I . The role of S‐nitrosoglutathione reductase (GSNOR) in human disease and therapy. Crit Rev Biochem. 2017;52(3):340‐354. 10.1080/10409238.2017.1304353 PMC5597050 28393572

[32] Tang CH , Seeley EJ , Huang X . Increased susceptibility to Klebsiella pneumonia and mortality in GSNOR‐deficient mice. Biochem Biophys Res Comm. 2013;442():122‐126. 24239886 10.1016/j.bbrc.2013.11.028 PMC3885541

[33] Lin H , Ma Y , Wei Y . Genome‐wide analysis of aberrant gene expression and methylation profiles reveals susceptibility genes and underlying mechanism of cervical cancer. Eur J Obstet Gynecol Reprod Biol. 2016;207:147‐152. 27863272 10.1016/j.ejogrb.2016.10.017

[34] Marchitti SA , Brocker C , Stagos D . Non‐P450 aldehyde oxidizing enzymes: the aldehyde dehydrogenase superfamily. Expert Opin Drug Metab Toxicol. 2008;4(6): 697–720. 18611112 10.1517/17425250802102627 PMC2658643

[35] Jackson B , Brocker C , Thompson DC . Update on the aldehyde dehydrogenase gene (ALDH) superfamily. Hum Genomics. 2011;5(4):283‐303. 21712190 10.1186/1479-7364-5-4-283 PMC3392178

[36] Weustenfeld M , Eidelpes R , Schmuth M . Genotype and phenotype variability in Sjögren‐Larsson syndrome. Hum Mutat. 2019;40(2):177‐186. 30372562 10.1002/humu.23679 PMC6587760

[37] Lee AY , Eri R , Lyons AB . CC chemokine ligand 20 and its cognate receptor CCR6 in mucosal T cell immunology and inflammatory bowel disease: odd couple or axis of evil? Front Immunol. 2013;4(undefined):194. 23874340 10.3389/fimmu.2013.00194 PMC3711275

[38] Christophi GP , Rong R , Holtzapple PG . Immune markers and differential signaling networks in ulcerative colitis and Crohn's disease. Inflamm Bowel Dis. 2012;18(12):2342‐2356. 22467146 10.1002/ibd.22957 PMC3407828

[39] Kaser A , Ludwiczek O , Holzmann S . Increased expression of CCL20 in human inflammatory bowel disease. J Clin Immunol. 2004;24(1):74‐85. 14997037 10.1023/B:JOCI.0000018066.46279.6b

[40] Ohtsuka Y , Sanderson IR . Dextran sulfate sodium‐induced inflammation is enhanced by intestinal epithelial cell chemokine expression in mice. Pediatr Res. 2003;53(1):143‐147. 12508094 10.1203/00006450-200301000-00024

[41] Kwon JH , Keates AC , Anton PM . Topical antisense oligonucleotide therapy against LIX, an enterocyte‐expressed CXC chemokine, reduces murine colitis. Am J Physiol Gastrointest Liver Physiol. 2005;289(6):1075‐1083. 10.1152/ajpgi.00073.2005 16099872

[42] Koukos G , Polytarchou C , Kaplan JL . A microRNA signature in pediatric ulcerative colitis: deregulation of the miR‐4284/CXCL5 pathway in the intestinal epithelium. Inflamm Bowel Dis. 2015;21(5):996‐1005. 25738378 10.1097/MIB.0000000000000339 PMC4402238

[43] Ishii KJ , Koyama S , Nakagawa A . Host innate immune receptors and beyond: making sense of microbial infections. Cell Host Microbe. 2008;3(6):352‐363. 18541212 10.1016/j.chom.2008.05.003

[44] Medzhitov R . Recognition of microorganisms and activation of the immune response. Nature. 2007;449(7164):819‐826. 17943118 10.1038/nature06246

[45] Berer K , Mues M , Koutrolos M . Commensal microbiota and myelin autoantigen cooperate to trigger autoimmune demyelination. Nature. 2011;479(7374):538. 22031325 10.1038/nature10554

[46] Citi S . Intestinal barriers protect against disease. Science. 2018;359(6380):1097‐1098. 29590026 10.1126/science.aat0835

[47] Arrieta MC , Finlay BB . The commensal microbiota drives immune homeostasis. Front Immunol. 2012;3(3):33. 22566917 10.3389/fimmu.2012.00033 PMC3341987

[48] Kaakoush NO , Day AS , Huinao KD . Microbial dysbiosis in pediatric patients with Crohn's disease. J Clin Microbiol. 2012;50(10):3258‐3266. 22837318 10.1128/JCM.01396-12 PMC3457451

[49] Papa E , Docktor M , Smillie C . Non‐invasive mapping of the gastrointestinal microbiota identifies children with inflammatory bowel disease. PLoS ONE. 2012;7(6):e39242. 22768065 10.1371/journal.pone.0039242 PMC3387146

[50] Morgan XC , Tickle TL , Sokol H . Dysfunction of the intestinal microbiome in inflammatory bowel disease and treatment. Genome Biol. 2012;13(9):R79. 23013615 10.1186/gb-2012-13-9-r79 PMC3506950

[51] Frank DN , St Amand AL , Feldman RA . Molecular‐phylogenetic characterization of microbial community imbalances in human inflammatory bowel diseases. Proc Natl Acad Sci U S A. 2007;104(34):13780–13785. 17699621 10.1073/pnas.0706625104 PMC1959459

[52] Shaw KA , Bertha M , Hofmekler T . Dysbiosis, inflammation, and response to treatment: a longitudinal study of pediatric subjects with newly diagnosed inflammatory bowel disease. Genome Med. 2016;8(1):1‐13. 27412252 10.1186/s13073-016-0331-y PMC4944441

[53] Osaka T , Moriyama E , Arai S . Meta‐analysis of fecal microbiota and metabolites in experimental colitic mice during the inflammatory and healing phases. Nutrients. 2017;9(12):1329. 10.3390/nu9121329 PMC5748779 29211010

[54] Flint HJ , Scott KP , Louis P . The role of the gut microbiota in nutrition and health. Nat Rev Gastroenterol Hepatol. 2012;9(10):577‐589. 22945443 10.1038/nrgastro.2012.156

[55] Lavelle A , Lennon G , O'Sullivan O . Spatial variation of the colonic microbiota in patients with ulcerative colitis and control volunteers. Gut. 2015;64(10):1553–1561. 25596182 10.1136/gutjnl-2014-307873 PMC4602252

[56] Chen W , Liu F , Ling Z . Human intestinal lumen and mucosa‐associated microbiota in patients with colorectal cancer. PLoS ONE. 2012;7(6):e39743. 22761885 10.1371/journal.pone.0039743 PMC3386193

[57] Jiang W , Wu N , Wang X . Dysbiosis gut microbiota associated with inflammation and impaired mucosal immune function in intestine of humans with non‐alcoholic fatty liver disease. Sci Rep. 2015;5:8096. 25644696 10.1038/srep08096 PMC4314632

