# Supplementary figures and images for: Taraxacum officinale extract ameliorates dextran sodium sulphate‐induced colitis by regulating fatty acid degradation and microbial dysbiosis
Source: J Cell Mol Med. 2019 Sep 29;23(12):8161–72. doi: 10.1111/jcmm.14686 (PMC6850927; doi:10.1111/jcmm.14686)

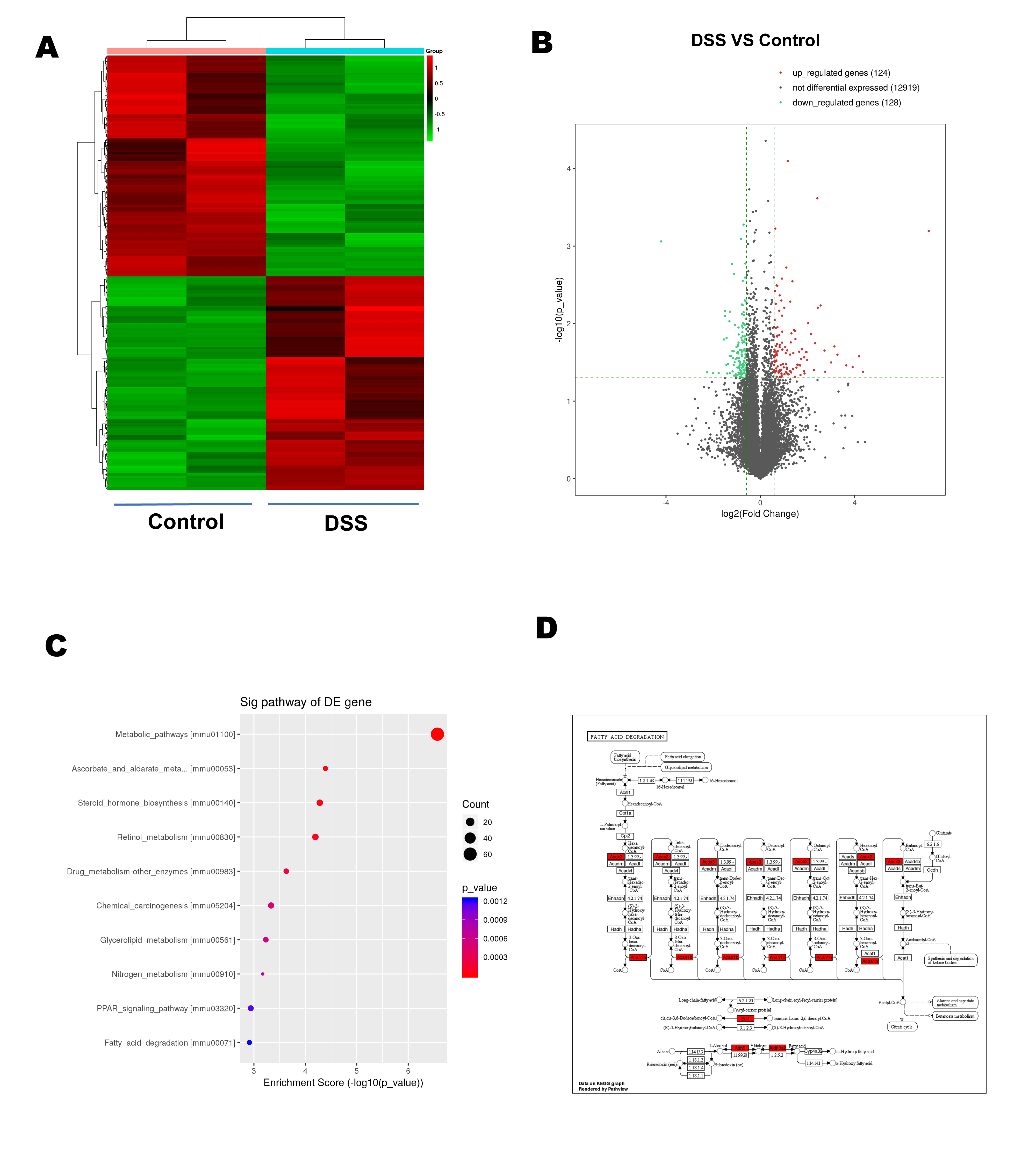

Supplement: Supplementary file 1 [file JCMM-23-8161-s001.tif]

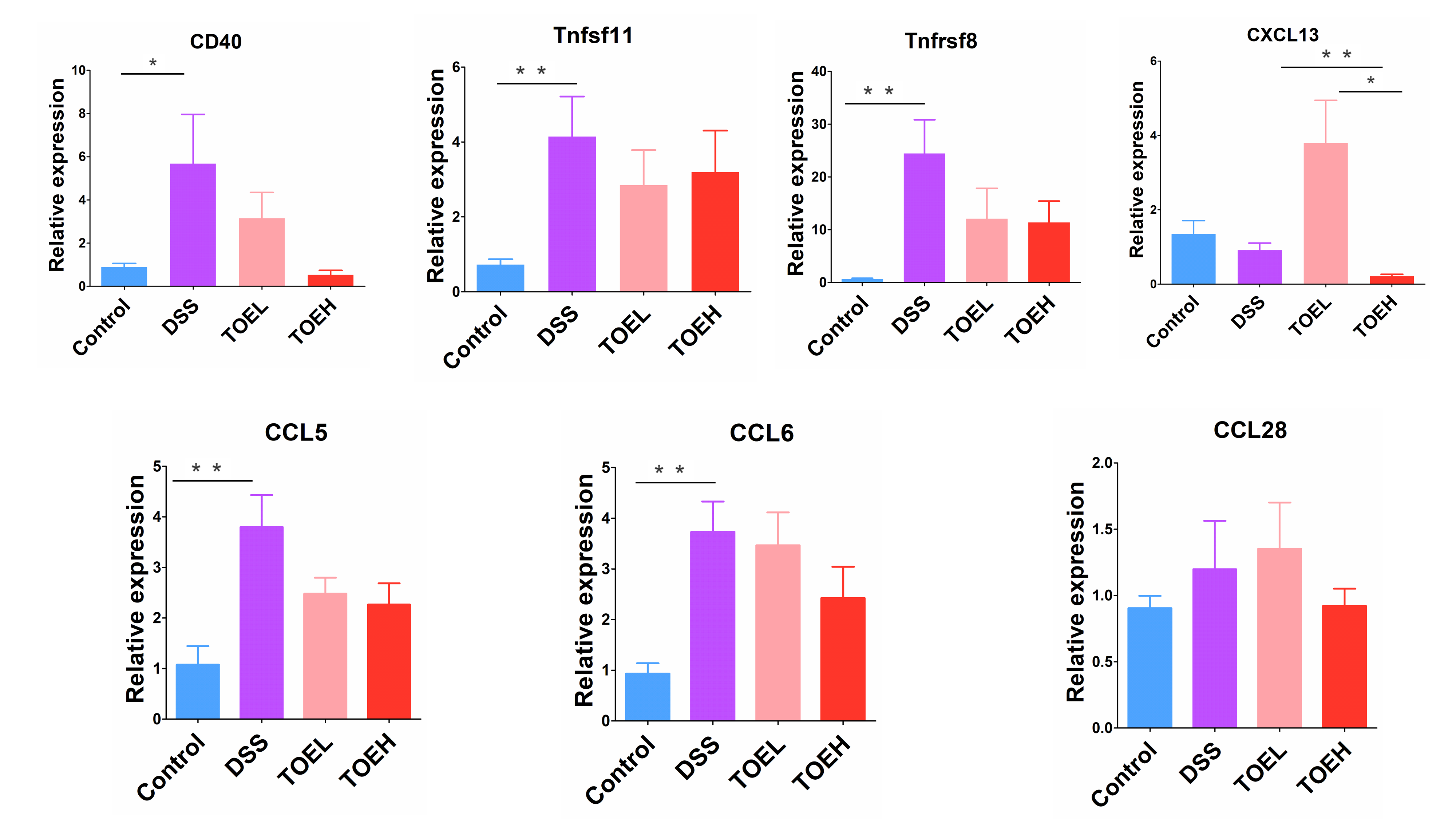

Supplement: Supplementary file 2 [file JCMM-23-8161-s002.tif]

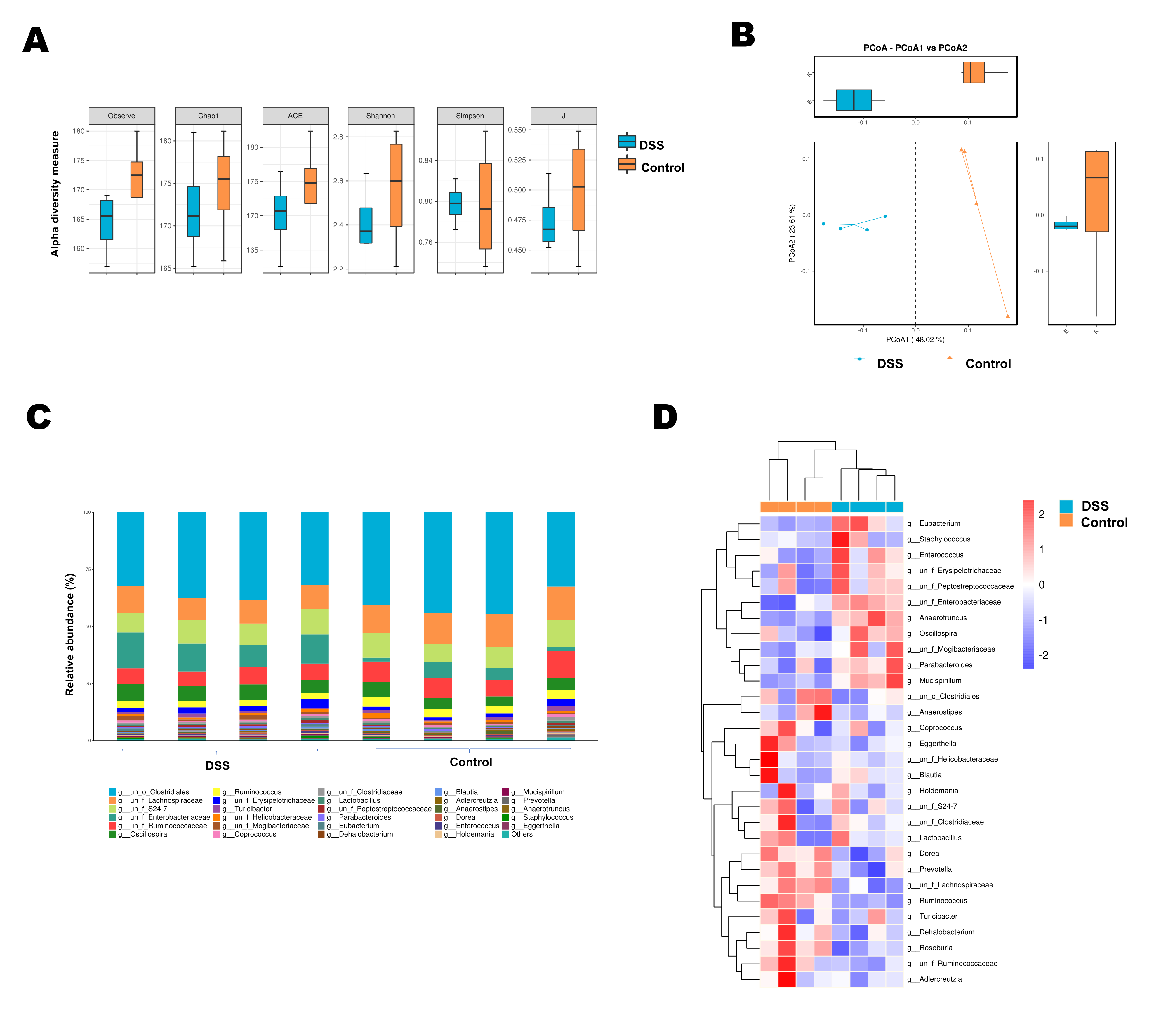

Supplement: Supplementary file 3 [file JCMM-23-8161-s003.tif]
