# Supplementary material for: Taraxacum officinale extract ameliorates dextran sodium sulphate‐induced colitis by regulating fatty acid degradation and microbial dysbiosis
Source: J Cell Mol Med. 2019 Sep 29;23(12):8161–72. doi: 10.1111/jcmm.14686 (PMC6850927; doi:10.1111/jcmm.14686)
Supplement: Supplementary file 5 [file JCMM-23-8161-s005.docx]

**Supplementary Figure legends**

**Supplementary Figure S1** The differentially-expressed genes were identified between DSS and control groups by transcriptome sequencing. **(A)** Hierarchical clustering analysis was performed to establish the gene expression profiling between these two groups. (**B)** Volcano plotting of the differentially-expressed genes between DSS and control groups. Red colors indicated the upregulated genes and blue colors indicated the downregulated genes. **(C)** KEGG analysis of the enrichment pathways of the differentially-expressed genes between DSS and control groups. (D) Schematic representation of the involvement of Aldh3a2, Acox3, and Adh5 in fatty acid degradation pathway.

**Supplementary Figure S2** The mRNA expression levels of CD40, Tnfsf11, Tnfrsf8, CXCL13, CCL28, CCL6 and CCL5 were determined by qRT-PCR analysis in control, DSS, TOEL and TOEH groups. * *P* <0.05, ** *P* <0.01.

**Supplementary Figure S3** Comparison of the gut microbial between DSS and control groups. **(A)** Alpha diversity was assessed by the Observe, Chao1, ACE, Shannon, Simpson and J index in DSS and control groups. **(B)** Beta-diversity was determined by the weighted PCoA in DSS and control groups. **(C)** Comparison of the difference in genus levels of gut microbiota between DSS and control groups. **(D)** The heat maps of the top 30 gut microbiota species in DSS and control groups.
